# Simultaneous Strain and Temperature Sensor Based on a Fiber Mach-Zehnder Interferometer Coated with Pt by Iron Sputtering Technology

**DOI:** 10.3390/ma11091535

**Published:** 2018-08-26

**Authors:** Xinran Dong, Haifeng Du, Xiaoyan Sun, Ji’an Duan

**Affiliations:** State Key Laboratory of High Performance Complex Manufacturing, College of Mechanical and Electrical Engineering, Central South University, 932 South Lushan Street, Changsha 410083, China; xrdong@csu.edu.cn (X.D.); duhaifeng@csu.edu.cn (H.D.); sunxy@csu.edu.cn (X.S.)

**Keywords:** Mach-Zehnder interferometer (MZI), multimode fiber (MMF), Pt coating, dual-parameter sensing

## Abstract

We demonstrated a fiber in-line Mach-Zehnder interferometer (MZI) coated with platinum (Pt) for the simultaneous measurement of strain and temperature. The sensor was fabricated by splicing a section of multimode fiber (MMF) between two single mode fibers (SMFs) and the Pt coating was prepared by iron sputtering technology. Fine interference fringes of over 20 dB with a compact size of 20 mm were achieved. The experimental results of the two different resonant dips showed strain sensitivities of −2.06 pm/με and −2.21 pm/με, as well as temperature sensitivities of 55.2 pm/°C and 53.4 pm/°C, respectively. Furthermore, it was found that the Pt coating can improve the strain sensitivity significantly, resulting in an increase of about 54.5%. In addition, the sensor has advantages of easy fabrication, low cost, and high sensitivity, showing great potential for the dual-parameter sensing of strain and temperature.

## 1. Introduction

Optical fiber sensors have been widely used in various fields such as structural engineering, health monitoring, chemical measurements, and aircraft due to its unique advantages of compact size, anti-electromagnetic interference, high sensitivity, and good compatibility [1,2]. In general, fiber sensors are sensitive to multiple physical variables, such as strain [3], bending, refractive index [4], and temperature [2]. To solve the cross-sensitivity issue of single-parameter sensing, multi-parameter fiber sensors have attracted the attention of many researchers. Recently, various sensing structures have been reported to achieve the simultaneous measurement of strain and temperature, such as: fiber Bragg gratings (FBGs) [5], long period gratings fabricated by CO_2_ laser [6], FBGs combined with four-core fiber [7], various fiber in-line Mach-Zehnder interferometers (MZIs) based on multimode fiber (MMF) [8] or plastic optical fiber [9], photonic crystal fiber (PCF) [10] or few-mode fiber [11], cascaded dual-pass MZIs with Sagnac interferometers [12], partially filled dual-core PCF [13] or selectively infiltrated PCF [14], dual-core As_2_Se_3_-PMMA (polymethyl methacrylate) tapers [15], and other structures [16,17,18,19]. Among them, fiber in-line MZIs have aroused great interest owing to their advantages of small size, simplicity of fabrication, and high sensitivity. The simultaneous measurement of strain and temperature can be realized by monitoring the spectral response of the different wavelengths. For example, in 2017, Sun et.al. [20] reported a fiber sensor by introducing higher order modes with twisted MMF based on single mode-multimode-single mode (SMS) structure for dual-parameter sensing. The obtained measurement resolutions reached up to ±2.14 με and ±0.89 °C, respectively. Furthermore, the strain sensitivity of the sensor was higher than that of the normal SMS structure. However, the sensor was realized by heating the MMF with an oxy-hydrogen flame generator and twisting the fiber at a certain rate. This method enhanced the complexity of fabrication and reduced the mechanical strength of the device. In 2016, Hou et al. [13] presented a multi-component interferometer based on partially filled dual-core PCF for the simultaneous measurement of strain and temperature. The sensor showed a temperature sensitivity as high as 5.43 nm/°C. However, the process of injecting liquid into the fiber core required accurate and sophisticated operations, which enhanced the complexity of fabrication, and the fiber materials increased the cost of financing. In addition, the fiber in-line MZIs based on SMF or MMF for the simultaneous sensing of strain and temperature are mostly combined with an FBG [21] or use a tapered structure [22]. However, introducing one FBG enhances the complexity of manufacturing and tapering the fiber shortens the life of the device. Moreover, their strain and temperature sensitivities needs to be further improved.

In this paper, a compact optical fiber MZI coated with Pt was proposed and experimentally demonstrated for the simultaneous measurement of strain and temperature. The MZI was fabricated by splicing an MMF with a length of 20 mm between two SMFs with a slight core-offset of about 7.5 μm, and the metal coating was prepared by an iron sputtering method. The experimental results demonstrated that the strain sensitivities of the MZI with Pt coating for two resonant dips were −2.06 pm/με and −2.21 pm/με in the range of 0~2000 με, respectively. Meanwhile, the corresponding temperature sensitivities of 55.2 pm/°C and 53.4 pm/°C were achieved in the range of 20 °C to 70 °C. Furthermore, the contrast experiments found that the Pt coating could enhance the strain sensitivity significantly. In addition, the sensor has many advantages including simple fabrication, low cost, and high fringe visibility, demonstrating its great application potential in the fields of health monitoring and structure quality monitoring.

## 2. Sensor Fabrication and Sensing Principle

### 2.1. Sensor Fabrication

**Figure 1:**
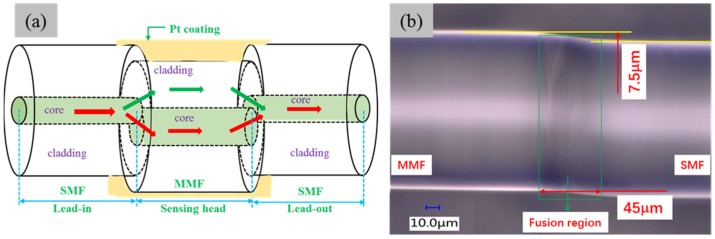
(**a**) Schematic diagram of the proposed sensor; (**b**) microscope image of the fusion region.

Figure 1a shows the schematic diagram of the proposed sensor. A 20-mm long MMF with a small core-offset was spliced between two SMFs using the attenuation mode (AM) in the commercial fusion splicer (FSM 80 s, Fujikura, Tokyo, Japan) menu. In Figure 1b, it can be observed that the offset between the core of the MMF and the SMF is about 7.5 μm and the width of the fusion region is about 45 μm. The core-offset section can inject the light into the cladding of MMF easily, exciting the higher order cladding modes. The core and cladding diameters of the SMF and MMF used in the experiment are 8.2 μm and 125 μm, 50 μm and 125 μm, respectively. The two SMFs act as lead-in and lead-out fibers, respectively, and the sensing head is the MMF. When the light passes through the lead-in fiber, the input light is divided into two parts at the core-offset fusion joints. One part transfers to the cladding of the MMF and the other propagates to the core of the MMF, playing the roles of cladding modes and the fundamental mode, respectively. At the second fusion joint, the light transmitted in the cladding will recouple into the core of the SMF and cause an interference pattern with the light transferred to the core of MMF, thus forming an MZI. This is due to the optical path difference caused by the refractive index (RI) difference between the fiber core and cladding. 

**Figure 2:**
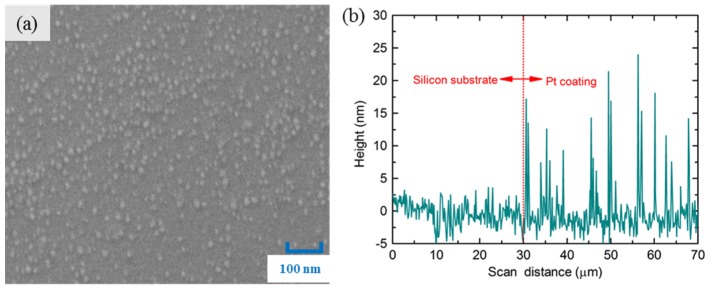
(**a**) SEM image of the Pt coating; (**b**) thickness of the Pt coating tested by atomic force microscopy (AFM).

The Pt coating was achieved using an ion sputtering coater (JFC-1600, JEOL Ltd., Tokyo, Japan), which can be well controlled merely by adjusting the splash time. This deposition technology is relatively simple and cheap compared with the chemical vapor deposition (CVD) method and atomic layer deposition (ALD) technology, as it does not require complex equipment or a sophisticated process control. To characterize the morphology and the average thickness of the Pt coating, Pt coating was sprayed on a silicon substrate with the same sputtering time of 200 s. The morphology of the Pt coating was observed by a scanning electron microscope (SEM), as shown in Figure 2a. It was found that the Pt particles attached to the surface of silicon, with diameters of about 10 ± 5 nm. The thickness of the Pt coating was tested by atomic force microscope (AFM, Dimension Icon, Veeco, NY, USA); the test probe scanned from the original silicon substrate area to the coating area in order to determine distance, and a height difference between the original substrate and the coating can be achieved, as shown in Figure 2b. It was found that the average thickness of the Pt coating was about 15 nm.

In addition, a broadband light source ranging from 1528 nm to 1602 nm and an optical spectrum analyzer (OSA, Agilent 86142B, Agilent Technologies, Santa Clara, CA, USA, wavelength range from 600 nm to 1700 nm) with a resolution of 10 pm were employed to trace the transmission spectrum evolution of the MZI during the fabrication and testing processes.

### 2.2. Sensing Principle

According to Figure 1, the phase difference between the cladding modes and fundamental core mode involved in the interference pattern can be expressed as follows [23]:

(1) $$\;\phi =\frac{2\pi \Delta n_{eff\;\;}L}{\lambda }$$

where $\Delta n_{eff}=n_{eff}^{core}-n_{eff}^{cl,m}$ denotes the effective refractive indices difference between cladding modes and the fundamental core mode; *λ* is the wavelength of the light; and *L* is the length of the MMF. According to Equation (1), the optimal interference fringes can be obtained under the condition of ϕ=(2m+1)π, and the resonant wavelength can be written as follows [23]:

(2) $$\;\lambda_{m}=\frac{2\Delta n_{eff\;}L}{2m+1}$$

where *m* is an integer. It can be seen from Equation (2) that the resonant wavelength is closely related to the length (*L*) and $\Delta n_{eff}$. When the sensor is subjected to strain, the fiber dimensions will be extended and the $\Delta n_{eff}$ will be changed due to the photo-elastic effect. Thus, a wavelength shift can be observed in the interference pattern, which can be expressed as shown below [22]:

(3) $$\;\frac{\Delta \lambda_{m}\;}{\lambda_{m}}=\left(1+\frac{p_{1}n_{eff}^{core}-p_{2}n_{eff}^{cl,m}}{n_{eff}^{core}-n_{eff}^{cl,m}}\right)\Delta \varepsilon$$

where $p_{1}$ and $p_{2}$ are the photo-elastic constants of the fiber core and cladding, respectively. Δε expresses the changes of strain. Further, when the sensor is heated, the change of temperature will cause changes in $\Delta n_{eff}$ and the length (*L*). So, the wavelength variation can be denoted as follows [21]:

(4) $$\;\frac{\Delta \lambda_{m}\;}{\lambda_{m}}=\left(\alpha +\frac{\xi_{1}n_{eff}^{core}-\xi_{2}n_{eff}^{cl,m}}{n_{eff}^{core}-n_{eff}^{cl,m}}\right)\Delta T$$

where α is the thermal expansion coefficients of the MZI; $\xi_{1}$ and $\xi_{2}$ are the thermo-optic coefficients of the fiber core and cladding, respectively. Δ*T* represents the change of temperature. When the sensor is heated, the change in the length of the MZI depends on the thermal expansion coefficients of the fiber and coating materials. Since the thermal expansion coefficient of the Pt coating is much higher than that of the silica, the effects of thermal expansion mainly depend on the coating. In general, the coating could cause changes in $n_{eff}^{cl,m}$ and α; thus, according to Equations (3) and (4), the resonant wavelength shift could occur when strain or temperature is applied to the sensor.

## 3. Experimental Results and Discussion

**Figure 3:**
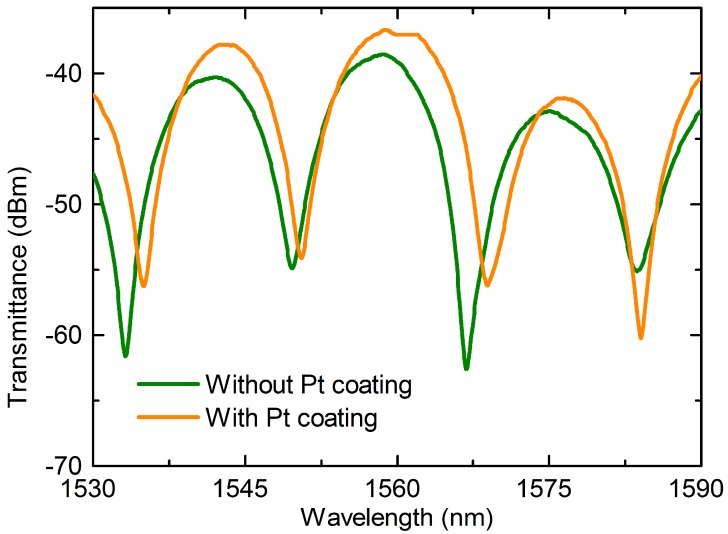
Transmission spectra of the Mach-Zehnder interferometer (MZI) without Pt coating and with Pt coating.

**Figure 4:**
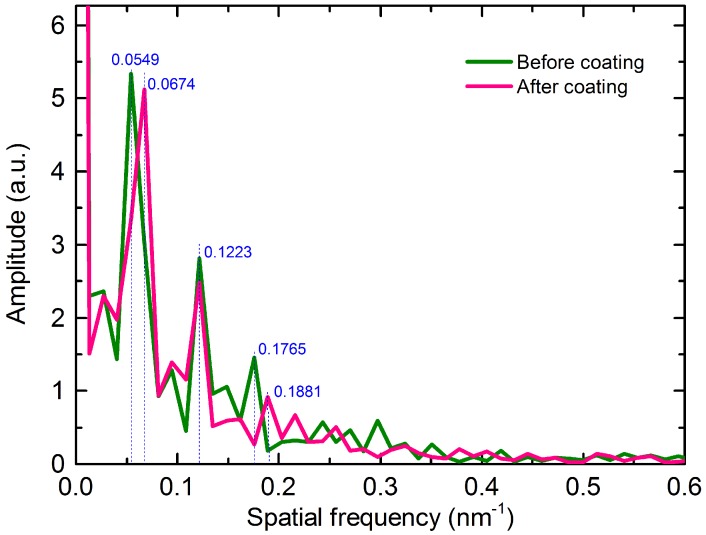
Spatial frequency of the MZI before coating and after coating.

Figure 3 shows the transmission spectra of the bare MZI and the MZI with Pt coating. As can be seen from the figure, the MZI experiences a slight red shift after the deposition of Pt. This occurs due to the change in the effective RIs of cladding modes in the presence of Pt. Meanwhile, the proposed MZI exhibits a fine fringe visibility as high as 23 dB and 20 dB before and after deposition of Pt, respectively. To analyze the number and the power distribution of the interference modes, the transmission spectra (illustrated in Figure 3) were achieved via fast Fourier transform (FFT) to obtain the spatial frequency spectra, as shown in Figure 4. It can be observed that there is one dominantly excited cladding mode for the MZI before coating and one after coating located at 0.0549 nm^−1^ and 0.0674 nm^−1^, respectively. This means that the order of dominant cladding modes excited in the MZI with Pt coating is higher than that without coating. So, the value of $n_{eff}^{cl,m}$ of the MZI after coating is relatively small. According to Equations (2) and (3), a red shift will occur after coating and the resonant dips will be more sensitive to the changes in strain.

The experimental setup for strain testing is illustrated in Reference [24]. The proposed sensor was mounted on two translation stages. The initial distance of the two stages was set as 20 cm and the strain was applied by moving the movable translation stage with a step of 50 μm. Meanwhile, a light source and an OSA were connected at the fiber ends to monitor the transmission spectra change. 

**Figure 5:**
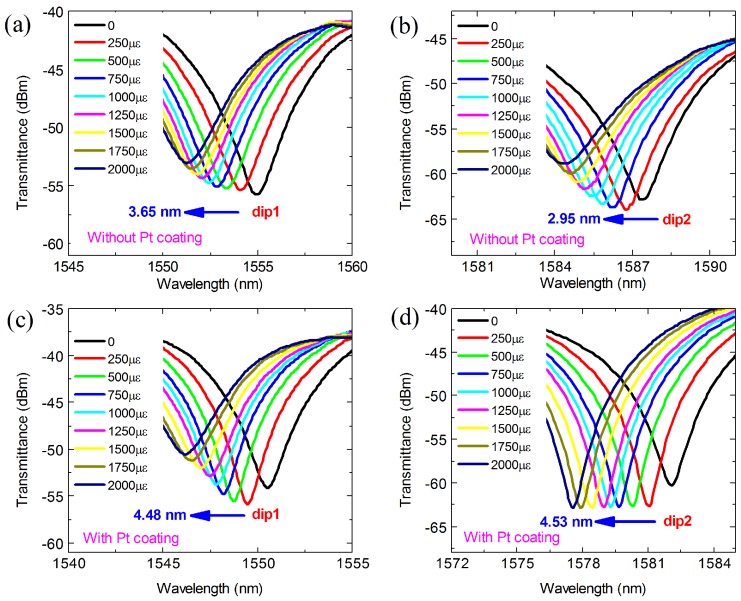
Transmission spectra response to strain of the MZI without Pt coating for (**a**) 1556 nm dip and (**b**) 1588 nm dip, and that with Pt coating for (**c**) 1551 nm dip and (**d**) 1582 nm dip.

**Figure 6:**
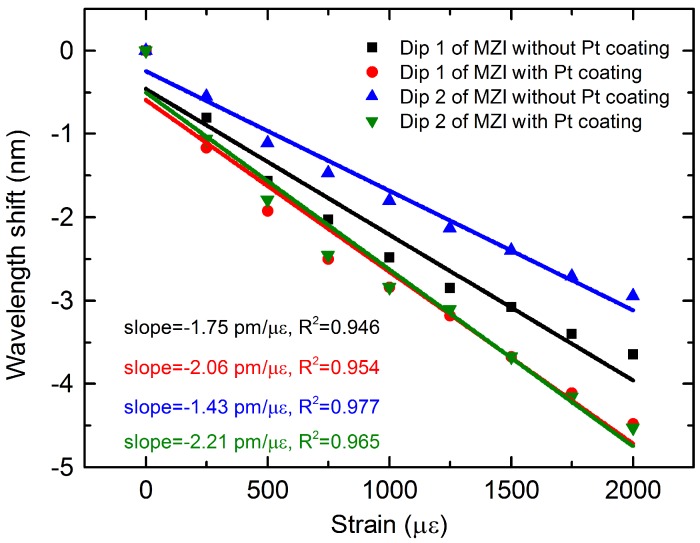
Relationship between the strain and wavelength shift of the MZI without Pt coating and that with Pt coating for two resonant dips.

The two dips of the MZI without and with Pt coating were chosen to analyze their strain and temperature sensing characteristics for the simultaneous measurement of strain and temperature. Figure 5 shows the transmission spectra response to strain of the MZI without and with Pt coating. It was found that all of the resonant dips shifted toward shorter wavelengths as the strain increased from 0 to 2000 με with a step of 250 με. For dip 1 and dip 2 of the MZI without Pt coating, the wavelength shifts of the corresponding dips were 3.65 nm and 2.95 nm, respectively, as described in Figure 5a,b. Those were significantly smaller compared to the results of the MZI coated with Pt, for which the corresponding wavelength shifts for dip 1 and dip 2 were 4.48 nm and 4.53 nm, respectively, as shown in Figure 5c,d. The wavelength shift response to the strain was linear, as shown in Figure 6. It was observed that the Pt coating can enhance the strain sensitivity obviously. Dip 1 and dip 2 of the MZI without Pt coating showed strain sensitivities of −1.75 pm/με and −1.43 pm/με, respectively, in the range of 0 to 2000 με. However, the strain sensitivities of the two corresponding dips of the MZI with coating were increased by 17.7% and 54.5%, up to −2.06 pm/με and −2.21 pm/με, respectively. The enhancement of the strain sensitivity is due to the greater changes in $\Delta n_{eff}$. The Pt coating excites the higher order cladding modes, thus the changes in RI of the cladding modes are greater compared to the MZI in air when strain is applied to the sensor. This means that more changes in $\Delta n_{eff}$, resulting in larger wavelength shifts and thus higher sensitivities, are achieved in the presence of Pt coating. In addition, the strain sensitivity we obtained was higher than that of the tapered SMS structure (0.7 pm/με) [22], the MZI based on polarization-maintaining PCF (1.01 pm/με) [25], and the traditional FBGs (1.4 pm/με) [26].

**Figure 7:**
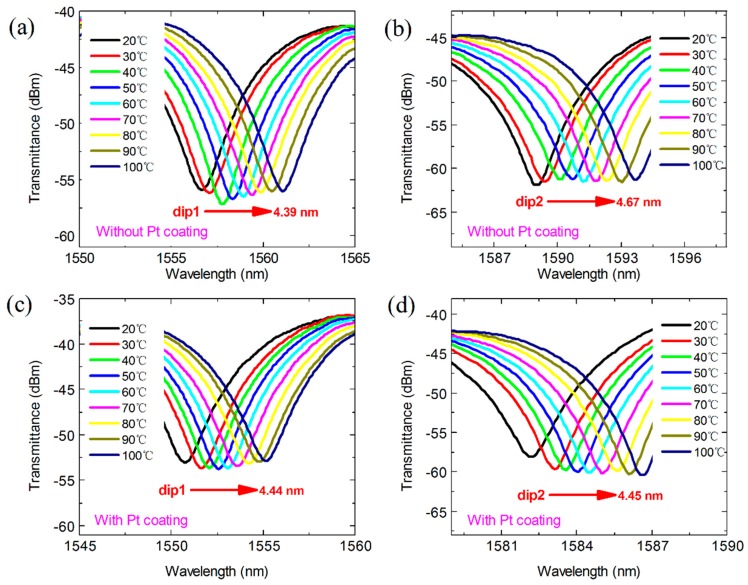
Transmission spectra response to temperature of the MZI without Pt coating for (**a**) 1556 nm dip and (**b**) 1588 nm dip, and that with Pt coating for (**c**) 1551 nm dip and (**d**) 1582 nm dip.

**Figure 8:**
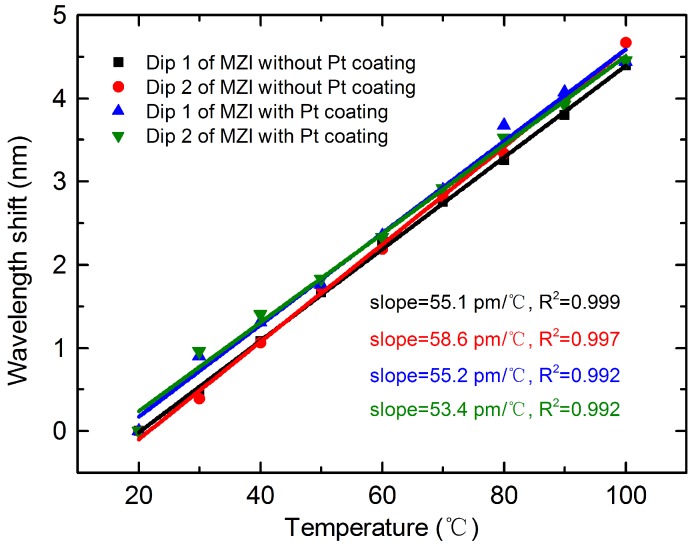
Relationship between the temperature and wavelength shift of the MZI without Pt coating and that with Pt coating for two resonant dips, respectively.

The temperature was controlled by using a heating plate, and the temperature changed from 20 °C to 100 °C with a step of 10 °C. With the increase of temperature, all the resonant dips exhibited a red shift, as shown in Figure 7. The wavelength shifts of the MZI without Pt coating for dip 1 and dip 2 were 4.39 nm and 4.67 nm, respectively, as shown in Figure 7a,b, in the range of 20 °C~100 °C. Their wavelength change was almost the same as those of the MZI coated with Pt, which were 4.44 nm at dip 1 and 4.45 nm at dip 2, as shown in Figure 7c,d. As illustrated in Figure 8, there is a linear relationship between the temperature and wavelength shift for the MZI without and that with Pt coating, with fitting linear correlation coefficients over 0.99. Furthermore, the temperature sensitivities for the MZI without Pt coating were 55.1 pm/°C at dip 1 and 58.6 pm/°C at dip 2. Meanwhile, temperature sensitivities of 55.2 pm/°C at dip 1 and 53.4 pm/°C at dip 2 for the MZI with Pt coating were achieved. From the above results, it can be seen that the Pt coating has a very small effect on the temperature sensitivity. This is because the melting point of Pt is greater than 1500 °C. However, the maximum applied temperature in our experiment was only 100 °C. The changes in the RI of the Pt coating caused by the increasing temperature were small; thus, the change of $\Delta n_{eff}$ can be neglected. Therefore, the wavelength shift of the MZI after coating was not obvious. 

When strain and temperature are simultaneous applied to the sensor, the wavelength shifts of dip 1 and dip 2 can be expressed using the standard matrix demodulation method, as reported in Reference [11]:

(5) $$\;\left[\begin{array}{c} \Delta \lambda_{1}\;\\ \Delta \lambda_{2} \end{array}\right]=\left[\begin{array}{cc} K_{1T} & K_{1\varepsilon }\\ K_{2T} & K_{2\varepsilon } \end{array}\right]\left[\begin{array}{c} \Delta T\\ \Delta \varepsilon \end{array}\right]$$

where $\Delta \lambda_{1}$ and $\Delta \lambda_{2}$ are the wavelengths of two dips, respectively; $K_{1T}$, $K_{2T}$ and $K_{1\varepsilon }$, $K_{2\varepsilon }$ are the temperature sensitivities and strain sensitivities of dip 1 and dip 2; ΔT and Δε are the temperature and strain variations. According to Equation (5), the temperature and strain measurement matrix can be expressed as follows [11]:

(6) $$\;\left[\begin{array}{c} \Delta T\;\\ \Delta \varepsilon \end{array}\right]=\frac{1}{D}\left[\begin{array}{cc} K_{2\varepsilon } & -K_{1\varepsilon }\\ -K_{2T} & K_{1T} \end{array}\right]\left[\begin{array}{c} \Delta \lambda_{1}\\ \Delta \lambda_{2} \end{array}\right]$$

where $D=|K_{1T}K_{2\varepsilon }-K_{1\varepsilon }K_{2T}|$ gives the absolute value of the determinant of the coefficient matrix.

According to the above experimental results and Equation (6), for the proposed MZI with Pt coating, the simultaneous measurement of strain and temperature can be achieved by calculating the sensitivity as below:

(7) $$\;\left[\begin{array}{c} \Delta T\;\\ \Delta \varepsilon \end{array}\right]=-\frac{1}{11.988}\times \left[\begin{array}{cc} -2.21 & 2.06\\ -53.4 & 55.2 \end{array}\right]\left[\begin{array}{c} \Delta \lambda_{1}\\ \Delta \lambda_{2} \end{array}\right]$$

According to this equation, the temperature and strain variation can be obtained by monitoring the wavelength shifts of two corresponding dips. According to the error analysis method given in Reference [11], if an optical spectrum analyzer (OSA) has a wavelength measurement resolution of δ(Δ*λ*_1_) and δ(Δ*λ*_2_) at two wavelengths, the theoretical strain and temperature resolutions of the sensor, δ(∆*ε*) and δ(∆*T*), can be given by [11]:

(8) $$\;\left[\begin{array}{c} \delta \left(\Delta T\;\right)\\ \delta \left(\Delta \varepsilon \right) \end{array}\right]=\pm \frac{1}{D}\left[\begin{array}{cc} \left|K_{2\varepsilon }\right| & \left|K_{1\varepsilon }\right|\\ \left|K_{2T}\right| & \left|K_{1T}\right| \end{array}\right]\left[\begin{array}{c} \delta \left(\Delta \lambda_{1}\right)\\ \delta \left(\Delta \lambda_{2}\right) \end{array}\right]$$

Since the OSA has a resolution of 10 pm, the temperature and strain resolution can be estimated to be about ±3.56 °C and ±90.59 με, respectively, according to Equation (8). 

## 4. Conclusions

In summary, an MZI coated with Pt was proposed for the simultaneous sensing of strain and temperature. The experimental results showed that the Pt coating could enhance the strain sensitivity obviously; the obtained sensitivities were increased by about 54.5%, from −1.43 pm/με to −2.21 pm/με. The simultaneous measurement of strain and temperature with the strain sensitivities of −2.06 pm/με and −2.21 pm/με, as well as temperature sensitivities of 55.2 pm/°C and 53.4 pm/°C, were realized through the demodulation matrices. Owing to its advantages of high sensitivity, low cost, fine fringe visibility of up to 20 dB, and simple manufacturing, the proposed sensor exhibited high potential for applications of physical sensing and structural health monitoring, etc.
